# Practitioners' Perceptions of the Soccer Extra-Time Period: Implications for Future Research

**DOI:** 10.1371/journal.pone.0157687

**Published:** 2016-07-06

**Authors:** Liam D. Harper, Melissa Fothergill, Daniel J. West, Emma Stevenson, Mark Russell

**Affiliations:** 1 Health and Life Sciences, Northumbria University, Newcastle-upon-Tyne, United Kingdom; 2 Institute of Cellular Medicine, Newcastle University, Newcastle-upon-Tyne, United Kingdom; Norwegian University of Science and Technology, NORWAY

## Abstract

Qualitative research investigating soccer practitioners’ perceptions can allow researchers to create practical research investigations. The extra-time period of soccer is understudied compared to other areas of soccer research. Using an open-ended online survey containing eleven main and nine sub questions, we gathered the perceptions of extra-time from 46 soccer practitioners, all working for different professional soccer clubs. Questions related to current practices, views on extra-time regulations, and ideas for future research. Using inductive content analysis, the following general dimensions were identified: ‘importance of extra-time’, ‘rule changes’, ‘efficacy of extra-time hydro-nutritional provision’, ‘nutritional timing’, ‘future research directions’, ‘preparatory modulations’ and ‘recovery’. The majority of practitioners (63%) either agreed or strongly agreed that extra-time is an important period for determining success in knockout football match-play. When asked if a fourth substitution should be permitted in extra-time, 67% agreed. The use of hydro-nutritional strategies prior to extra-time was predominately considered important or very important. However; only 41% of practitioners felt that it was the most important time point for the use of nutritional products. A similar number of practitioners account (50%) and do not (50%) account for the potential of extra-time when training and preparing players and 89% of practitioners stated that extra-time influences recovery practices following matches. In the five minute break prior to extra-time, the following practices (in order of priority) were advocated to players: hydration, energy provision, massage, and tactical preparations. Additionally, 87% of practitioners advocate a particular nutritional supplementation strategy prior to extra-time. In order of importance, practitioners see the following as future research areas: nutritional interventions, fatigue responses, acute injury risk, recovery modalities, training paradigms, injury epidemiology, and environmental considerations. This study presents novel insight into the practitioner perceptions of extra-time and provides information to readers about current applied practices and potential future research opportunities.

## Introduction

Soccer is an intermittent team sport, requiring periods of both low- and high- intensity activity, and skill execution. Soccer matches are typically played as two 45 min halves, however; an additional 30 min is required (termed extra-time; ET) when a match requires an outright winner and scores are level after 90 min. Between 1986 and 2014, 35% of senior FIFA World Cup matches have required ET, including the last three finals. The requirement for ET in soccer tournaments is becoming more prevalent, with 50% of knockout matches at the 2014 FIFA World Cup requiring 120 min of match-play when compared to 25% at the 2002 and 2010 FIFA World Cup’s, and 38% at the 2006 competition.

Research into the responses to ET is in its infancy despite the volume of literature examining the demands of soccer match-play [1–3]. Recently, authors have indicated that ET has a negative impact on both technical [4] and physical [5] performances. For example, Harper et al. [4] observed reductions in the total number of passes and the number of successful passes and dribbles during ET relative to 90 min. Moreover, during an English Premier League reserve team cup match, with data derived from 10 Hz GPS units, Russell et al. [5] observed reductions in total distance covered, high intensity distance covered, number of sprints and the total number of accelerations and decelerations. As these are important aspects of successful soccer performance [6], it appears that ET has negative implications for players.

Contemporary qualitative research involving professional soccer practitioners has investigated the use of training load and player monitoring [7], warm-up practices [8], and injury prevention strategies [9, 10]. Developing a deeper understanding of how applied practitioners operate in a professional environment can allow researchers to better appreciate the complexities involved, and conduct research that is both pertinent and effective. Drust & Green [11] suggest a theoretical model which is similar to that of Bishop [12] implying that researchers should investigate the aetiology of the problem, conduct descriptive research and gain an understanding into the possible barriers preventing uptake, while undergoing studies to test the effectiveness of an intervention and its possible implementation in an applied setting.

Consequently, it is important to understand the perceptions and consequences of ET for applied practitioners, and the factors that hinder applied practice and intervention application. This can then be subsequently followed by studies investigating the efficacy of a particular intervention and its transferability to the applied setting. Therefore the aim of this study was to assess practitioner perceptions of ET through qualitative measures (i.e., the use of an open ended online survey). This was to gather information on their opinions of ET, their current practice, and possible future research areas thereby providing scientific researchers with more detailed information regarding the consequences of ET for future research.

## Methods

Following ethical approval from the Health and Life Sciences Ethics Committee at Northumbria University, 120 practitioners from national and international soccer teams were identified as having roles associated with the preparation and recovery practices of elite soccer players. Practitioners were contacted either electronically (*n* = 55) or *via* postal letter (*n* = 65) between July and September 2015. Only one practitioner per soccer team was contacted. We requested only one response per team to ensure the findings were not influenced by multiple responses from the same team. Completed responses were returned by staff from 46 individual teams, representing a 38% response rate; a rate which is higher or similar to previous qualitative research involving soccer practitioners [7–10].

Participant information was provided before the survey and each practitioner gave consent before study involvement. The survey was created using an online resource (Bristol Online Surveys, University of Bristol, UK) with an approximate completion time of 10 min. All responses were anonymous, with practitioners not being required to disclose their name or affiliation, but only their role, competitive level, and general location (Tables 1 and 2). The survey contained eleven main questions with nine sub questions in a scaled, rank or open ended format (see S1 File for full survey). The unstructured or open ended component allowed practitioners to expand upon and provide further detail with regards to their own ET practice and experiences. The desired elaboration of specific points was encouraged by activating a feature that required a conscious button click to progress to the next question.

**Table 1:** Practitioner role within their professional soccer team.

Role	*n*
Sport Scientist	21
Fitness Coach	9
Strength and Conditioning Coach	4
Sport Performance Manager	4
Physiotherapist	3
Athletic Trainer	2
Team Doctor	1
Exercise Physiologist	1
Head of Science and Medicine	1

**Table 2:** League and competitive level of practitioners.

League and Level	*n*
English Premier League Academy	6
English Premier League Senior	5
English Championship Senior (second tier)	4
English League One Senior (third tier)	4
English League Two Senior (fourth tier)	4
Major League Soccer	4
Italian Serie A Senior	3
International Team Senior (country unspecified)	3
Academy Level (location unspecified)	3
French Ligue 1 Senior	2
Elite Level (location unspecified)	2
International A Squad	1
Liga MX (Mexico) Senior	1
Australian A-League Senior	1
Eredivisie Senior (Netherlands)	1
English League One Academy	1
Danish Superliga Academy Team	1

### Survey topics

#### ET and match success

Practitioners stated how much they agreed with the following question: ‘*extra-time is an important period for determining success in football match play*’ by using a 5-point Likert-type scale with the following options given: *strongly agree*, *agree*, *neither agree or disagree*, *disagree*, *strongly disagree*.

#### Current ET regulations

Practitioners views regarding the use of a fourth substitution during ET (as per FIFA discussions at International Football Association Board meetings held in February 2015; www.fifa.com) were ascertained by their yes or no responses to the question ‘FIFA are currently considering allowing a fourth substitution during extra-time. Do you believe this is warranted?’

#### Nutritional interventions and ET

Practitioners’ perceptions of the effectiveness of hydro-nutritional ET interventions were assessed by level of perceived importance using a 5-point Likert-type scale (*very important*, *important*, *somewhat important*, *not important*, *not sure*). The current use of any particular nutritional supplementation strategy before or during ET was assessed with opportunity for practitioners to elaborate on the specifics of the intervention or to provide further details about why no particular hydro-nutritional interventions were employed. The perceived importance of the use of nutritional products prior to ET relative to other time points (i.e., pre-match or half-time) was also assessed.

#### Research paradigms

To determine if the practitioners felt that more research should be conducted into ET, the level of importance (*very important*, *important*, *somewhat important*, *not important*, *not sure*) of the following research areas was investigated: *fatigue responses*, *nutritional interventions*, *training paradigms*, *recovery modalities*, *environmental considerations*, *injury epidemiology*, and *acute injury risk*. Practitioners were also given the opportunity to suggest other areas or factors they felt were important which had not been mentioned directly whilst those who selected *not important* were able to give their reasons why.

#### Current Preparation and Training Practices

Practitioners specified what they did differently or adapted if an upcoming match had the potential to go to ET when training and preparing their players, or to give reasons for not making any adjustments.

#### Current Match-Day Practices

To examine the influence of the potential for ET on match-day practices when compared to matches of only 90 min duration, practitioners were asked to specify any modifications to practice or provide reasoning as to why usual procedures remained unchanged. In order of importance, respondents ranked what they advocated to players during the five min break separating the cessation of normal time and the beginning of ET from the following options: *energy provision*, *hydration*, *massage*, *tactical preparations*, or *other* (which they were asked to specify). Practitioners also stated the applicability of these strategies in the two minute break between the 15 min halves of ET, stating which were the most important or why they were not applicable.

#### Current Post-Match Practices

To investigate the influence of ET on recovery modalities and training prescription, the practices performed in the immediate (e.g., same day) and prolonged (e.g., +24 and +48 hour) periods following a match requiring ET was explored. Practitioners were provided with an opportunity to state what changes were made, if any, or to elaborate on the decision to not modify practices following ET involvement. Practitioners also proposed a list of recommendations that they would make to recovery strategies if given the opportunity.

#### Data Analysis

The study design is of a cross-sectional and descriptive nature and as such, the data is presented in a descriptive manner. For questions with categorical responses, points for the level of importance were awarded as thus: ‘*very important*’– 3 points, ‘*important*’– 2 points, ‘*somewhat important*’– 1 point, ‘*not sure*’– 0.5 points, ‘*not important*’– 0 points, as per McCall et al. [10]. Thereafter, total accumulated points were calculated and answers ranked in order of highest to lowest. For questions utilising a Likert-scale, frequency analysis was used to establish the percentage of practitioners who had endorsed a particular response. For the question regarding what practitioners advocated prior to ET (i.e., question 8a on supplementary materials), the option rated first was awarded 5 points, second—4 points, third—3 points, fourth—2 points, and fifth—1 point. Points were then collated to determine mean order of importance.

Written responses for the open-ended questions were read several times for familiarisation and to develop a full understanding of the content [13]. The raw data were then organised and subjected to inductive content analysis by the lead author (LH). Inductive analysis is a data driven technique which occurs independently of any pre-existing frameworks or preconceptions [14]. Similar emergent themes were classified as general dimensions and assigned a descriptive overarching label. Second order themes were then established and inductive analysis continued until data saturation had occurred. In view of the lead author’s prior knowledge of the research area, the research team employed independent validation of the themes at every stage of analysis. Moreover, peer debriefing and member checking (a form of independent validation) was also employed by the research team to enhance credibility and ensure that an accurate representation of the data had occurred [15]. In the final stages of analysis a deductive approach was employed to confirm the validity of the inductive analysis and to establish any theoretical relationships within the data [14].

## Results

From the inductive and deductive analysis, seven general dimensions emerged from the data with regards to practitioners’ views on ET, and their current or ideal ET preparation. These were ‘importance of ET’, ‘rule changes’, ‘efficacy of ET hydro-nutritional provision’, ‘nutritional timing’ ‘future research directions’, ‘preparatory modulations’, and ‘recovery’.

### Importance of ET

The majority of practitioners (63%, *n =* 29) either agreed or strongly agreed that *extra-time is an important period for determining success in football match play* whereas 30% (*n =* 14) neither agreed nor disagreed, with the remaining 7% of respondents (*n =* 3) disagreeing with this statement.

### Rule changes

Notably, 67% (*n* = 31) of practitioners felt that a fourth substitution should be allowed in ET; however, 33% (*n* = 15) felt that current substitution rules (i.e., three substitutes throughout whole match duration) should remain. Three second order themes were identified for practitioners in favour of a fourth substitute: fatigue (e.g., *‘it will help to improve performance and reduce fatigue’)*, injury risk (e.g., *‘allow for any injuries and to decrease load related injuries in future training and games’*), and tactical modifications (e.g., *‘may make for differing team strategies’*).

Conversely, support for current rulings to remain was explained by three second order themes, namely: lack of evidence of risk (e.g., *‘I don’t think extra time poses a ‘risk’ to players who have played the full match and so I am unsure what the rationale for a fourth substitute is; if they want to avoid meaningless periods of play due to fatigue*, *reintroduce the golden goal concept’*), players are fit enough (e.g., *‘players are able to complete 120 minutes’*), and preparedness (e.g., *‘the better conditioned teams should be rewarded for their hard/smart work’*).

### Efficacy of ET hydro-nutritional provision

Practitioners were asked about the effectiveness of hydro-nutritional ET interventions; with none stating hydro-nutritional ET interventions were not important. The majority (89%, *n* = 41) believed that they are either very important or important and 11% (*n* = 5) found them somewhat important.

### Nutritional Timing

When asked whether the use of nutritional products prior to ET was more important than at any other time point, 59% (*n* = 27) of practitioners disagreed whereas 41% (*n* = 19) agreed. The following second order themes were identified as reason for it not being the most important time point: similar importance (e.g., *‘I think it is equally as important as both prior to kick off and at a half time*. *The players must be fully nourished the whole time’*), lack of benefit/evidence (e.g., *‘timeframe probably too short to have any real physical impact—probably more psychological*), and other time points are more important (e.g., *‘no*, *I believe the food consumption 24–48 hours prior to match-play has the greatest impact on performance’*).

Fatigue (e.g., *players are in a state of fatigue prior to extra time and physiologically and psychologically require an energy boost*), and diminished energy supply prior to ET (e.g., *‘glycogen depletion might influence performance more after 90 min of play’*) were identified as second order themes regarding why the break before ET is the most important time point for ingestion of nutritional products.

### Future research directions

**Fig 1:**
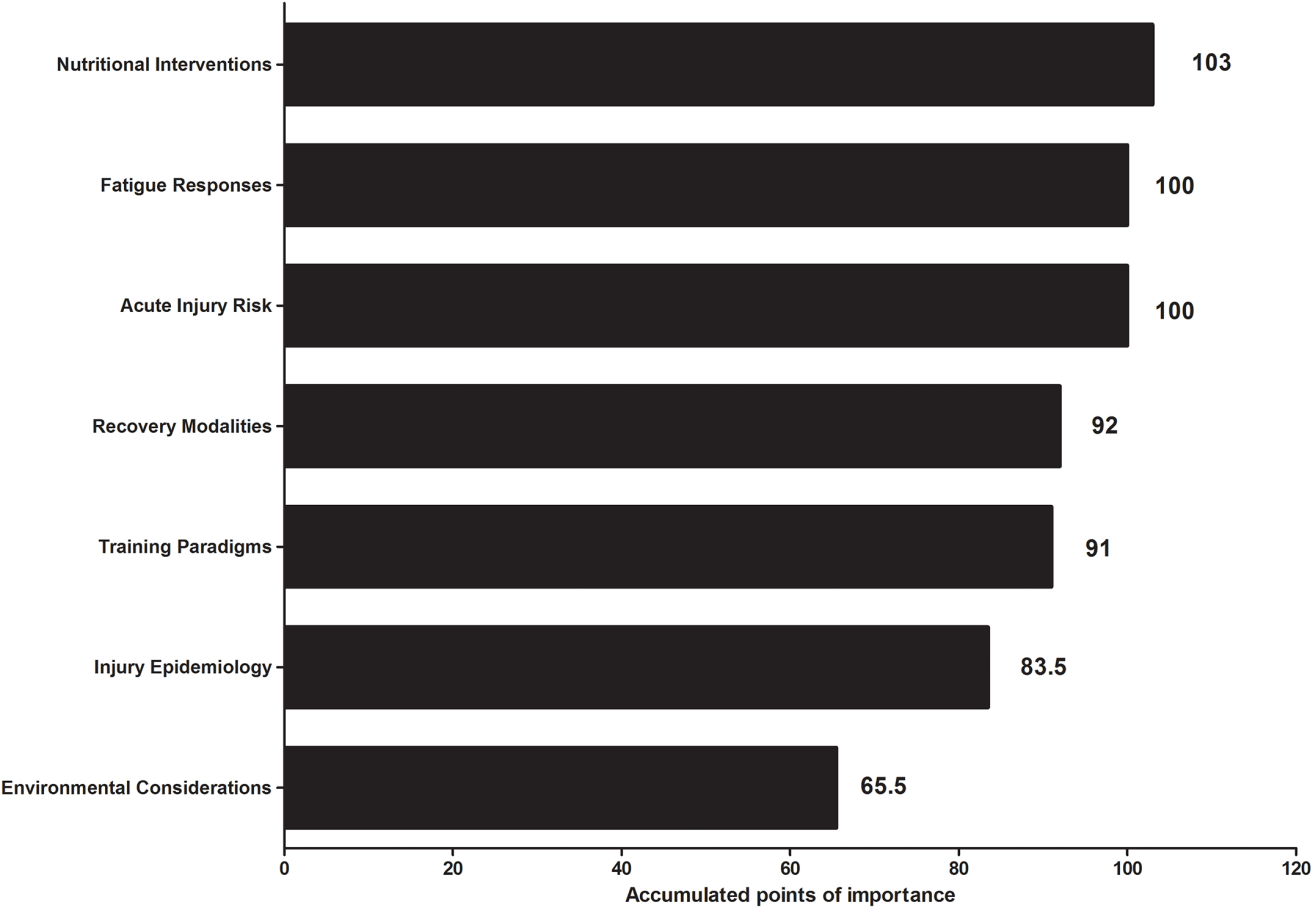
Accumulated points of importance for future extra-time research areas.

Respondents were mostly positive towards the need for further research into ET (i.e., 91%, *n =* 42) with only 9% (*n* = 4) of practitioners disagreeing with this statement. Second order themes were identified for disagreement as: scarcity (e.g., *‘happens too infrequently—other bigger issues more important’*), and no issues (e.g., *‘I don’t perceive extra time as an issue for players well prepared’*). The accumulated points of importance for potential research areas are given in Fig 1, with nutritional intervention studies viewed as the most important, followed by, in order of importance, research into fatigue responses, acute injury risk, recovery modalities, training paradigms, injury epidemiology, and lastly, environmental considerations. Six respondents gave other areas that were not mentioned but which they felt were important. When these areas were grouped into second order themes, psychological (e.g., *‘does the team with momentum at the end of 90 minutes continue into the extra time period’*), and performance outputs (e.g., *‘alterations in physical markers during this period*, *for example*, *m/min’*) emerged.

### Preparatory Modulations

A similar number of practitioners account (50%, *n* = 23) and do not (50%, *n* = 23) account for the potential of ET when training and preparing their players. The second order themes that we identified relating to the changes made to preparations are provided in Table 3.

**Table 3:** Second order themes (*bold italicised*) with quotes to support why or why not changes are made to pre-match preparations.

**Changes that are made pre-match**
***Training modulations***: *‘adapt training in build up to a fixture that may go into extra time’; ‘reduce training intensity/duration 2 days prior to match’; ‘taper*, *with reduction in volume 2–3 days pre-match’; ‘additional fitness or small-sided games added on to end of training’*
***Nutritional adjustments***: *‘additional food strategy used (supplements)’*
***Player education***: *‘before games with the potential of extra-time*, *players are informed of the importance of hydration and calorie intake’*; *‘ensure players follow a structured post training recovery and nutrition strategy prior to the game’*
**Why changes are not made pre-match**
***Injury risk*: *‘****incorporating training with a view to maximizing performance in the extra time period year round (as a fixture requiring extra time could occur at any stage of the season depending on success) would result in higher loads and increased injury risk’*
***Scarcity*:** *‘too infrequent’; ‘does not occur frequently enough to be considered as a pertinent training variable to address’; ‘it is not a frequent enough occurrence to warrant increased workload throughout the season’*
***Insufficient time to improve fitness*:** *‘playing every 3 days limits ability to develop fitness—players rotation and management is key’; ‘unlikely a single microcycle would be enough to induce required adaptations’*
***Priorities*:** *‘focus on league games—may not even be involved in any extra time in a whole season’; ‘majority of games we play are 90 minutes and we base training loads around 90 minute games’*
***Unnecessary*:** *‘players are overloaded so specific training and preparation is not needed’; ‘a fit group should be capable to cope with around 1 or 2 matches per season’*

When asked if they revised or adapted their current match-day practice in light of the potential of ET, 67% (*n* = 31) of practitioners reported no changes were made whilst 33% (*n* = 15) stated that changes were made. Six second order themes emerged regarding practitioners’ match day practices. Hydro-nutritional adjustments (e.g., *provide more fuel sources*, *i*.*e*., *carb/caffeine gels’)* and player management (e.g., *‘consider squad rotations more closely’*) were two second order themes related to changes that practitioners make to their preparatory practices. Infrequency (e.g., *‘only small chance of extra time’*), normal time being of greater importance (e.g., *‘we prepare to win the game in regulation as we would any game’*), preparedness (e.g., *‘we prepare for the game as per normal*, *players should be optimally fuelled and hydrated in preparation for any game*), and negative impact of change (e.g., *‘we treat each match with the same preparation as to not upset the normal pre-match routine of our squad’*) were recognised as four second order themes for not modifying current preparatory practices.

**Fig 2:**
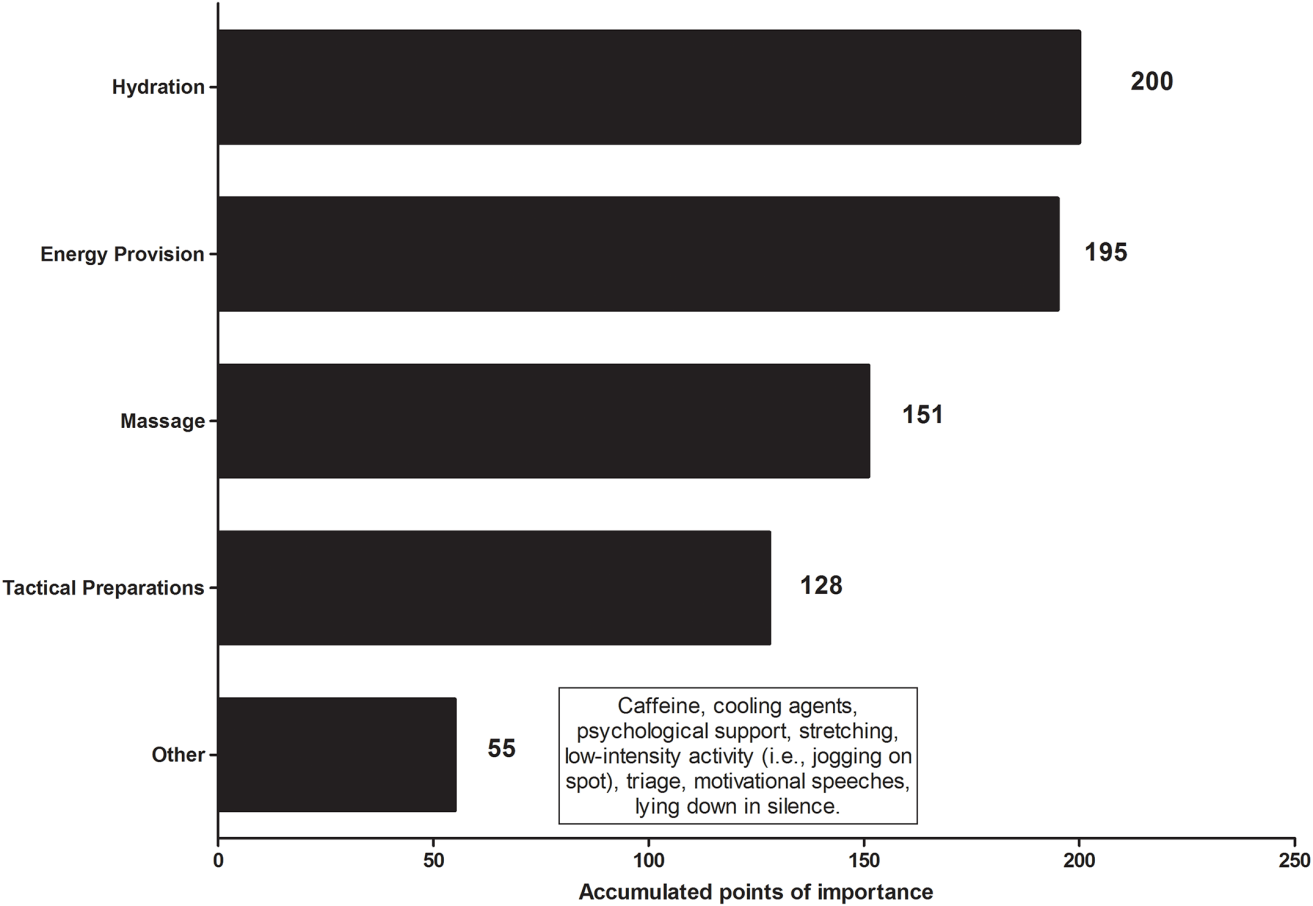
Accumulated points of importance for what practitioners advocate to players in the five min break prior to extra-time.

**Fig 3:**
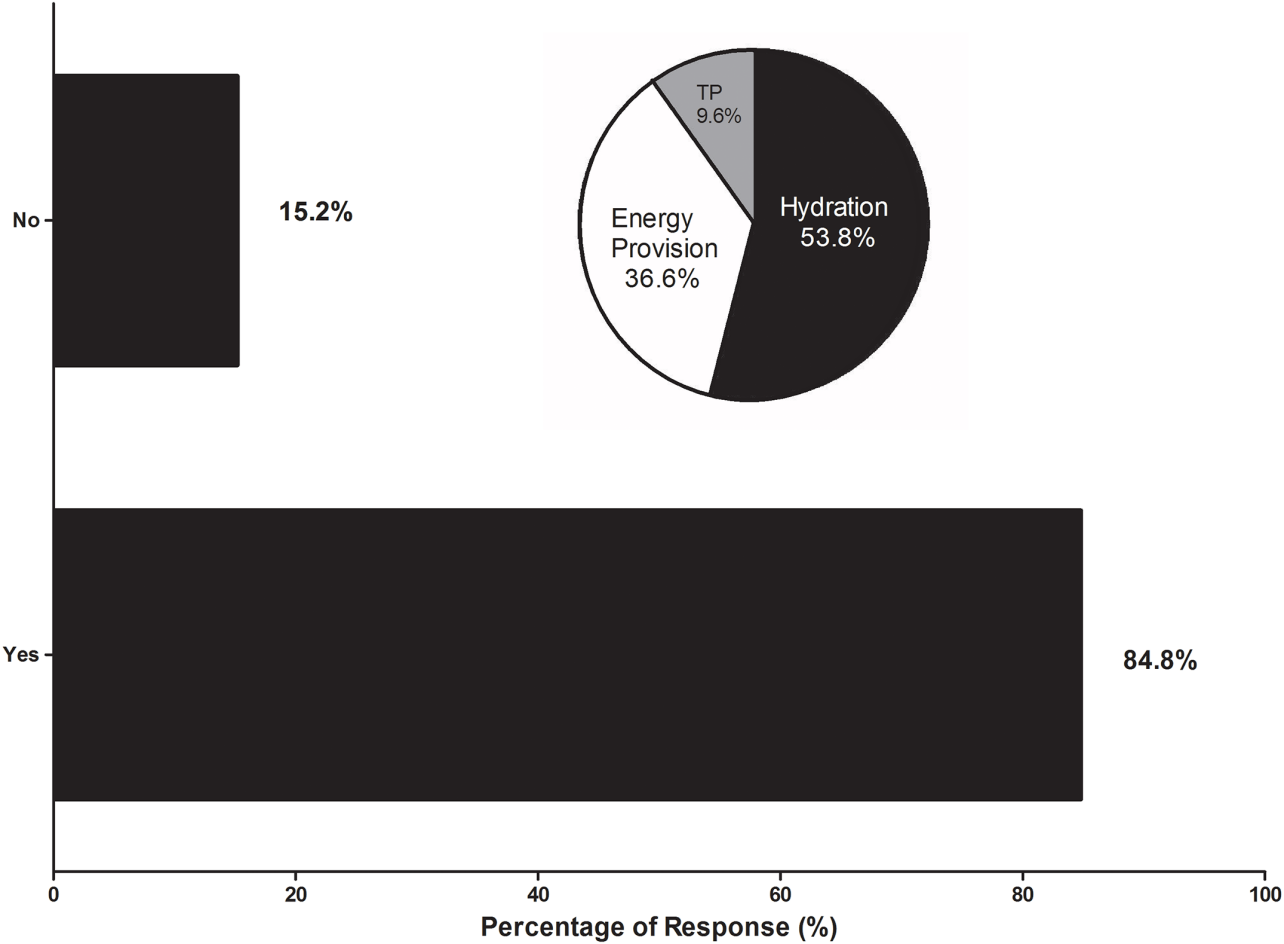
Percentage of practitioners who do or do not advocate a particular strategy in the break between the two halves of extra-time. Pie chart depicts what practitioners advocate by percentage. TP = tactical preparations.

Fig 2 shows the accumulated points of importance of what practitioners typically advocated to their players in the five minute break between the end of 90 min and the beginning of ET. Hydration was identified as the most important, followed by energy provision, massage, tactical preparations and other practices. Other practices advocated to players are listed on Fig 2. Furthermore, 85% (*n* = 39) of practitioners felt that the two min break between the two halves of ET was an opportunity to apply the strategies stated above, with hydration (54%), energy provision (37%) and tactical preparations (10%) seen as the most applicable at this time point (Fig 3). Additionally, 87% (*n* = 40) of practitioners advocate a particular nutritional supplementation strategy prior to ET. We identified two second order themes: *hydration* (e.g., *‘electrolytes’*; *‘water’*), *energy provision* (e.g., *‘high CHO drinks or gels’*; ‘*high glycemic index snack–jelly beans’*; *‘caffeine’*; *‘protein’*). Second order themes identified from the 13% (*n* = 6) who do not recommend anything in particular prior to ET were: efficacy (e.g., *‘not enough time to take effect’*), and habitual (*‘continue using the same players train with and have used already in the game’*).

### Recovery

Notably, 89% (*n* = 41) of practitioners stated that ET influences the recovery practices in the immediate and prolonged periods following a match, with 11% (*n* = 5) stating that no changes are made. Only one second order theme for not changing recovery was identified: current system (e.g., *‘post-match recovery strategies are implemented following the same method as league matches’*). The changes that practitioners make are given in Table 4, along with a list of changes related to recovery that they would make if given the choice.

**Table 4:** Second order themes (*bold italicised*) with quotes to support why or why not changes are made to recovery following matches requiring extra-time.

**Changes to recovery practices following matches that require extra-time**
***Training modulations*:** *‘training adapted to wellness scores*, *countermovement jump performance*, *ankle mobility*, *hip internal rotation range of motion*, *hamstring mobility*, *and adductor strength scores’; ‘split squad into 2*^*nd*^*/3*^*rd*^ *days with reduced intensity’; ‘late start to training match day +1 for extra sleep’*
***Recovery modifications*:** *‘all recovery modalities become compulsory’; ‘mandatory ice baths*, *foam rolling and massage’; ‘additional time spent promoting flexibility/mobility work for 72 hours post-match’*
***Nutritional adjustments*:** *‘increased carbohydrate and protein provision’; ‘increased carbohydrate consumption 0–48 hrs post match’*
**Changes to recovery strategies if given the choice**
***Training modulations***: *‘additional passive rest days to allow players to be unloaded’; ‘reductions in training load 48hrs post match’*
***Nutritional adjustments*:** *‘increased intake of recovery promoting foods’; ‘cooked meal in the dressing room’; ‘increased carbohydrate provision’*
***Recovery modalities*:** *‘pool based recovery—post game + following day’; ‘access to hydrotherapy immediately following match’; ‘mandatory ice baths’*
***Monitoring tools*:** *‘increased fatigue monitoring of individuals’; ‘extra markers such as blood*, *saliva and sleep would be interesting to monitor more closely’*

## Discussion

The aim of this study was to assess the perceptions of professional soccer practitioners concerning the ET period. This included understanding current and ideal practices and opinions on ET, as well as elucidating future research areas. This is the first study to investigate perceptions of ET in this population and as such provides novel findings in the emerging area of ET research.

The majority of practitioners (63%) felt that ET is an important period for determining success in a soccer match and as such they feel they may be able to influence match outcome. A high number of domestic and international cup competitions stipulate in the rules of competition that a 30 min ET period must be played if matches are tied at 90 min (www.fifa.com; www.uefa.com). Between 1986 and 2014, 35% of senior FIFA World Cup knockout matches have required ET, including the last three finals. In the annually held English League Cup (LC) competition, 23% of matches required ET from August 2011 to February 2015. Notably, 39% (*n* = 18) of our sample are practitioners working for teams involved in this competition. All LC matches (except the final) are played midweek, with league matches played on the preceding and succeeding Saturday or Sunday. Furthermore, knockout matches played in major international tournaments such as the FIFA World Cup or UEFA European Championships are typically separated by 72 h. If matches are of normal duration (i.e., 90 min), this may still have implications for recovery as congested fixture periods have been shown to increase injury risk [16], and physiological stress [17], and diminish some aspects of tactical [18], and physical performance [19]. Notably, 89% of the practitioners sampled in this study changed their recovery strategies following a match requiring ET, with second order themes identifying: training modulations, recovery modifications and nutritional adjustments (Table 4).

No research currently exists investigating the influence of a match requiring ET on recovery and subsequent performance in matches of close temporal proximity. As indices of technical and physical performance have been shown to reduce in ET [4, 5], it would seem logical that recovery may also be compromised, however; this supposition remains to be investigated. A number of recovery strategies that practitioners would like to utilise in their applied setting are also given in Table 4 and include monitoring tools and recovery modalities. Notably, recovery modalities are considered the fourth most important future research area (Fig 1). A number of recovery strategies purported to accelerate recovery and improve soccer performance have been examined (for a review see [20]). However, the effectiveness of these strategies for recovery from a match requiring ET has yet to be investigated.

Practitioners were predominately positive about the use of nutritional interventions prior to ET and future research on their effectiveness. Indeed, 89% of practitioners felt that hydro-nutritional interventions in the five min break between 90 min and ET were very important or important, with the remaining 11% finding them somewhat important. A large majority (87%) of practitioners recommend a particular nutritional supplementation strategy to their players prior to ET. Recommendations include hydration and energy provision. However, evidence supporting the use and efficacy of nutritional interventions during ET are lacking, with only one study to date observing ingestion of 46 g of carbohydrate in the 5 min break prior to ET improves dribbling performance, but not indices of physical performance [21]. Furthermore, imbibing carbohydrate-electrolyte solutions during exercise has also been shown to elongate time to fatigue during intermittent shuttle running following a 90 min soccer-specific simulation [22]. The acute benefits of carbohydrate supplementation on skill performance [23], intermittent exercise performance [24] and prolonged periods of exercise [25] are well known. Further investigations are required to optimise hydro-nutritional strategies for players engaging in ET, especially as the practitioners we sampled believe nutritional intervention studies are the most important area for future research (Fig 1).

The use of caffeine as an ergogenic aid is popular [26], however; there are equivocal results regarding its effectiveness during simulated [27, 28] and actual soccer match-play [29, 30]. Some practitioners recommend caffeine prior to ET, however; the effectiveness of such a strategy is yet to be researched. Peak serum caffeine concentrations are typically observed 30–45 min post-ingestion; therefore the full ergogenic benefit of ingesting caffeine prior to ET may not be achieved. However, caffeinated gum, which stimulates caffeine absorption quicker than capsule form [31], may provide a more efficient method of ingestion. Nonetheless, caffeine ingestion prior to ET may possibly compromise post-match sleep quality [32] especially as ET periods are predominately played at night, with some matches finishing as late as 23:30 h.

The majority of practitioners felt that the two min break between the two halves of ET was an opportunity to implement strategies to improve performance (see Fig 3). However, the two min break is typically seen as the only opportunity for teams to switch halves, without returning to the technical area, therefore making it difficult to implement any particular strategy. From anecdotal observations, the actual length of time for the break tends to vary. Indeed, during the FIFA World Cup in Brazil in 2014 the players were given an extended break lasting ~5 min. Practitioners should be cognisant of the fact that it may not be possible to always gain access to the players during this break, as it would seem to be under the referee’s discretion. As hydration and energy provision are seen as the main strategies used, practitioners should place hydro-nutritional products in the goalmouth or close to pitch-side for the players to readily use.

Half of practitioners modified pre-match practices to account for the potential of ET. Currently, no data exists that has examined the influence of training modalities on performance during ET. Implementing specific training microcycles to try and acutely enhance adaptations may be difficult, with barriers including injury risk and insufficient time to improve fitness (Table 3). Although in-season twice weekly high-intensity interval training has been shown to improve maximal aerobic speed and 40 m sprint velocities [33], this training was performed over a 10 week period. The acute benefits are unlikely to be obtained if the training is performed only in the week leading up to the match. Changes that practitioners make related to training are varied, with some reducing volume and some providing an extra exercise stimulus (training modulations; Table 3).

Practitioners make changes to diet (nutritional adjustments; Table 3) and emphasise good nutritional habits (player education; Table 3) in the days prior to a match that may require ET. Adequate energy intake relative to match demands is considered important [34], with insufficiencies potentially leading to negative effects on performance [35]. Therefore, as it is likely players will expend more energy during ET than during 90 min; energy intake in the days prior to the match must be optimised. The majority (67%) of practitioners did not change any of their usual match-day practices on a match-day that may involve ET. Currently, little evidence exists regarding the influence of ET on performance or the aetiology of fatigue. As 91% of practitioners feel that future research should be conducted on ET, with fatigue responses as the second most important area (Fig 1), researchers should consider avenues to further investigate this period of play.

The influence of ET on acute injury risk has yet to be investigated, with practitioners considering it the joint second most important research area (Fig 1). Epidemiological and quantitative data suggest that there is a greater risk of muscular injury during the last 15 min of a 90 min match, with comprised lower body strength output and movement mechanics postulated as potential mechanisms [36–38]. Furthermore, a passive half-time period has been demonstrated to increase hamstring injury risk [39]. Future research is required to assess acute injury risk during ET as well as following the passive 5 min break that precedes ET. Furthermore, epidemiological studies, similar to the UEFA Champions League study [16], are required to both assess the incidence and type of injury sustained in ET, and the influence of ET on injury incidence during periods of fixture congestion.

A rule change to allow a fourth substitute in ET has been proposed on a number of occasions by FIFA. Indeed, in March 2016 the International Football Association Board advisory panel sanctioned the trialling of a fourth substitute at the 2016 Olympic games in Rio de Janeiro, the FIFA Under-20 Women’s World Cup 2016 in Papua New Guinea and the FIFA Club World Cup 2016 in Japan. The majority of practitioners (67%) were in favour of the introduction of a fourth substitute, with fatigue, injury risk and tactical modifications identified as second order themes. Further research is required to ascertain the influence of an additional substitute on player health, performance, and match outcome.

Environmental considerations were considered the least important of the seven suggested future research areas (Fig 1). This may be due to the location of the practitioners sampled (i.e., predominately from temperate climates close to sea level). However, the negative influence of the environment (i.e., temperature and altitude) on soccer performance has been demonstrated previously [40, 41]. With FIFA World Cup tournaments (i.e., Qatar 2022, Brazil 2014, South Africa 2010), and other major matches (Champions League final 2014, Berlin, Germany; 26°C, and UEFA European Championship final 2012, Kiev, Ukraine; 26°C) potentially played at altitude or in the heat, the health risks and performance effects of such conditions during ET require investigation.

Although other approaches have been used for qualitative data analysis (e.g., grounded theory; and narrative analysis), the general inductive approach used presently has been demonstrated as a simple, robust method to analyse qualitative data [13]. It is prudent to note when invited to partake in the investigation, practitioners were made aware of the topic (i.e., ET). We therefore acknowledge that the respondents who did not complete the survey may not have had an interest in ET, possibly skewing our findings. Furthermore, practitioners completed the survey during the months of July, August, and September and as such, their team may not have yet been exposed to ET in that competitive season. However, it is likely they had been exposed to ET in previous seasons; therefore their present circumstances were unlikely to influence their responses.

## Conclusions

In summary, this study presents a novel insight into practitioner perceptions of the soccer ET period. We provide evidence demonstrating that the majority of practitioners feel that ET is an important period of match-play for success and endorse the use of a fourth substitution in ET. Furthermore, we have provided information regarding what research practitioners consider should be conducted in future, and how they currently prepare and recover players participating in ET. Not only could coaches and practitioners use this information to inform current practices, researchers could use this information to develop innovative research projects to both better understand the influence of ET on performance, but also intervention strategies that could be tested and then applied to the target population (i.e., professional soccer players).

## Supporting Information

S1 File Survey Questions. (XLSX)
